# Spotlight on NLRP6 and Tumor Research Situation: A Potential Cancer Participant

**DOI:** 10.1155/2023/6613064

**Published:** 2023-06-28

**Authors:** Liangzheng Chang, Yuying Tian, Lei Xu, Qiuyao Hao, Lingyu Song, Yinying Lu, Yunhuan Zhen

**Affiliations:** ^1^Guizhou Medical University, Guiyang, Guizhou, China; ^2^Inner Mongolia Medical University, Hohhot, Inner Mongolia, China; ^3^Department of Gastroenterology, Affiliated Hospital of Guizhou Medical University, Guiyang, Guizhou, China; ^4^Comprehensive Liver Cancer Center, The Fifth Medical Center of PLA General Hospital, Beijing 100039, China; ^5^Department of Colorectal Surgery, Affiliated Hospital of Guizhou Medical University, Guiyang 550004 Guizhou, China

## Abstract

NOD-like receptor family pyrin domain containing 6 (NLRP6) is a new pattern recognition receptor in the mammalian innate immune system. Both the liver and the gut exhibit substantial levels of cytoplasmic expression. It can speed up cell response to endogenous danger signals or exogenous pathogen infection. NLRP6 can function in various ways as an inflammasome or a noninflammasome. The understanding of NLRP6 is steadily increasing thanks to ongoing investigations, but due to discrepancies in how those studies have described their link with tumors, the significance of NLRP6 in the emergence of cancer is still debatable as of this writing. This article will use the structure and function of NLRP6 as the pivotal point and thoroughly explain the present interactions between NLRP6 and tumors and any possible clinical benefits.

## 1. Introduction

Tumor immunology is increasingly developing into a hot region for people to investigate the occurrence, development, and treatment of cancers as tumor cognition becomes more sophisticated. The role of the immune system in cancer has not been fully understood in recent years because tumors can effectively suppress the immune response by activating negative regulatory pathways (also known as checkpoints) related to immune homeostasis or by adopting features that enable them to actively evade detection [1]. Recent decades have seen a tremendous advancement in people's understanding of immunology, which has led to the gradual emergence of treatment approaches utilizing this perspective. Currently, CAR-T cell therapy and immune checkpoint inhibitors targeting the PD-1/PD-L1 (programed cell death protein 1/programed cell death ligand 1) pathway have been successful in the clinical treatment of T-cell-centered tumor immunity [2–4]. However, the current T-cell-centered tumor immunotherapy is only effective in a small number of cancer patients. According to earlier research, the innate immune response is necessary for the establishment of long-term protective memory T cells as well as the occurrence and maintenance of the T-cell response [5]. It is critical to increase understanding of innate immunity in cancer given the critical function that innate immune response plays in the immune system.

**Figure 1:**
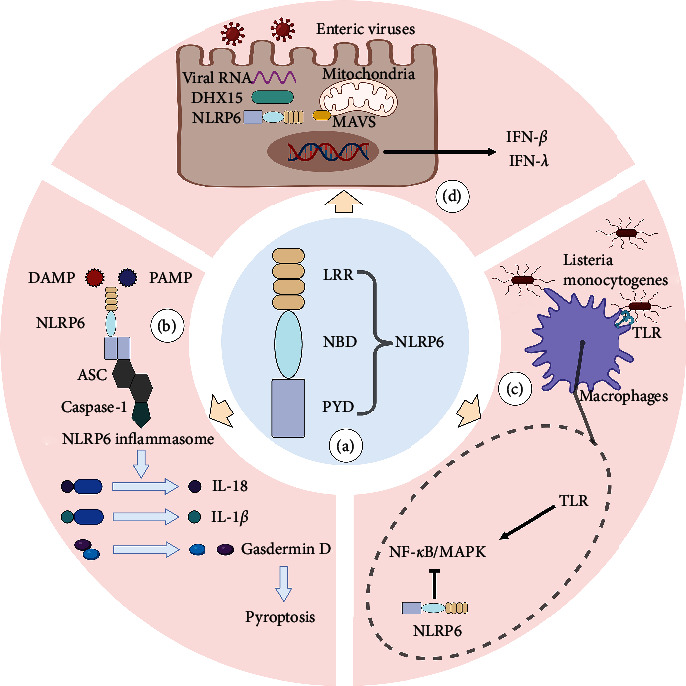
The structure and representative function of NLRP6. (a) The basic structure of NLRP6 is composed of N-terminal PYD, central NBD, and C-terminal LRR domain. (b) After recognizing PAMP and DAMP, NLRP6 assembles with ASC and Caspase-1 to form NLRP6 inflammasome, which promotes the maturation of pro-IL-1*β* and pro-IL-18 and gasdermin-D-induced pyroptosis. (c) NLRP6 interferes with the activity of NF-*κ*B and MAPK in mouse macrophages after bacterial infection. (d) NLRP6 binds to viral RNA through DHX15 and triggers the induction of I/III IFN through MAVS. PYD, pyrin domain; NBD, nucleotide-binding domain; LRR, leucine-rich repeat; PAMP, pathogen-associated molecular pattern; DAMP, damage-associated molecular pattern; ASC, apoptosis-associated speck-like protein; TLR, Toll-like receptor; NF-*κ*B, nuclear factor kappa B; MAPK, mitogen-activated protein kinase; MAVS, mitochondrial antiviral signaling protein; DHX15, DEAH-box helicase 15.

Innate immunity refers to a collection of inherent defense mechanisms that species eventually evolve throughout the course of phylogenetic development. Through pattern recognition receptors (PRR), such as pathogen-associated molecular patterns (PAMP) and damage-associated molecular patterns (DAMP), the host of mammals detects a variety of harmful infections and endogenous dangerous signals, thereby inducing an innate immune response [6]. The nucleotide-binding domain and leucine-rich repeat-containing family (NLR) is a group of PRR present in the cell that helps the cell to detect the source of pressure and infection [7]. According to the N-terminal domain, NLR can be divided into four subfamilies: the acidic transactivation domain (NLRA), the baculoviral inhibitory repeat-like domain (NLRB), the caspase activation and recruitment domain (CARD; NLRC), and the pyrin domain (NLRP) [8]. According to their roles, the known NLRs can be broadly classified into three groups. First, a subset of NLRs recognizes PAMP and DAMP and then forms an inflammasome, a polymer–protein complex. In the course of the innate immune response, the inflammasome can control the activation of caspase-1 or caspase-11 and encourage the maturation and release of cytokine precursors pro-IL-1 and pro-IL-18 [9]. Under pathological inflammatory and stress conditions, the inflammasome can also regulate caspase-1-dependent programed cell death—pyroptosis and trigger cell death [10]. Furthermore, several NLRs participate in the transmission and control of common intracellular signaling pathways, including the nuclear factor-*κ*B (NF-*k*B) and mitogen-activated protein kinase (MAPK) pathways, which influence the production of cytokines and chemokines downstream [11]. Additionally, NLRs can boost antiviral effects and control immune responses to viruses [12]. However, it is interesting to note that NLRP6 is the only NLR member discovered thus far to be active in each of these processes [7] (Figure 1).

The relationship between cancer and chronic inflammation is well-known today [13]. There is significant epidemiological evidence that nonsteroidal anti-inflammatory medicines, particularly aspirin, are effective chemoprophylaxis against cancer, and chronic inflammatory illnesses can raise the risk of various malignancies. Second, because the tumor microenvironment contains a lot of inflammatory cells and mediators, reducing these elements in relevant animal models can have a big impact on how quickly cancer develops, spreads, and metastasizes. Therefore, the prevention and therapy of malignant diseases can be aimed at this intricate inflammatory network [14]. Inflammation is also acknowledged as one of the cancer markers because it plays a role in the growth and development of cancer as well as its treatment and recovery [15]. A key driver of carcinogenesis, which promotes gene mutation, tumor formation, and progression, is chronic inflammation brought on by aberrant NLR signal transduction [16]. It is more important for us to continue researching NLRP6, a novel form of NOD-like receptor, as the importance of NLRs in cancer becomes more apparent [17]. Beginning with NLRP6's fundamental structure, this paper explains the NLRP6 inflammasome's assembly method, the state of NLRP6 research, and the various roles that NLRP6 plays in malignancies of associated tissues and organs. It also covers NLRP6's possible clinical use.

## 2. NLRP6: A Novel NLR Family Member

### 2.1. Structure of NLRP6 and the NLRP6 Inflammasome

NOD-like receptor family pyrin domain containing 6 (NLRP6) is a recently identified member of the NLRs. It was formerly known as PYPAF5 [18]. Grenier's study further opened the door to NLRP6 research by demonstrating that the coexpression of NLRP6 and apoptosis-associated speck-like protein (ASC) synergistically stimulated the NF-*κ*B signaling pathway as well as caspase-1-dependent cytokine processing [19]. In terms of structure, NLRP6 is made up of a C-terminal leucine-rich repeat domain (LRR), a central nucleotide-binding domain (NBD), and an N-terminal pyrin domain (PYD) (Figure 1(a)). Similar to other NLRs with comparable structural features, NLRP6's LRR and NBD domains exhibit a closed conformation and self-inhibition in the absence of ligands, which can successfully block the self-activation of NLRP6 inflammasome [20]. When the ligand conjugates, NBD and LRR adopt an open conformation, and the ASC^PYD^ filaments come together to form a nucleation thanks to the filaments created by more NLRP6^PYD^ [21]. ASC functions as a supramolecular platform for caspase-1 recruitment and activation. Through card-card interaction, it initiates caspase-1 CARD filaments, activating caspase-1 [22]. The maturation of pro-IL-1 and pro-IL-18, as well as Gasdermin-D-induced pyroptosis, are all facilitated by activated Caspase-1, allowing cells to react swiftly to endogenous danger signals or external pathogen invasion (Figure 1(b)).

### 2.2. NLRP6 Inflammasome Activation

There are various layers that control how the NLRP6 inflammasome expresses itself. Peroxisome proliferator-activated receptor *γ* (PPAR-*γ*) agonists and microbial signals of type I interferon (IFN) can be used as efficient ways to mediate NLRP6 transcription at the level of transcriptional regulation [23, 24]. There are numerous activators and regulators at the assembly level of the NLRP6 inflammasome. For instance, mouse macrophages can bind to lipoteichoic acid, which is the primary component of Gram-positive bacteria's cell walls, and stimulate the assembly of the NLRP6 inflammasome [25]; NLRP6 inflammasome regulation by microbial metabolites can be positive or negative. The mouse NLRP6 inflammasome is activated by taurine generated from bile acid, while it is inhibited by histamine and polyamine spermine [26]; enzyme for deubiquitin Cyld controls the development of IL-18 by deubiquitinating NLRP6 and preventing the establishment of the NLRP6-ASC inflammasome complex [27]. NLRP6 and double-stranded RNA (dsRNA) interact to cause liquid–liquid phase separation (LLPS), which establishes a new research avenue for the development of the NLRP6 inflammasome [28].

Of course, NLRP6 is also independently involved in several studies in addition to helping the inflammasome develop and function. For instance, NLRP6 inhibits NF-*κ*B and MAPK activity in mice macrophages following bacterial infection and decreases neutrophil recruitment [29] (Figure 1(c)). NLRP6 is primarily expressed in the digestive system, including the small intestine, large intestine, and stomach, but it is also significantly expressed in organs outside of the digestive system, including the liver, spleen, and lung, as well as neurons, lymphocytes, and bone marrow-derived cells [30]. It is, therefore, not difficult to see why the majority of NLRP6 research studies are concentrated on the aforementioned organs and cells and why these studies also corroborate NLRP6's involvement in the aforementioned organs and cells' associated cancers (Table 1).

## 3. Role of the NLRP6 in Cancer

### 3.1. NLRP6 in Colorectal Cancer

Of all malignancies, colorectal cancer (CRC) has one of the highest incidence and fatality rates [45]. Increased vulnerability to CRC can result from colonic inflammation brought on by infections and wounds [46]. It is believed that environmental influences, such as “ecological dysbiosis,” or changes in gut flora, as well as host genetic variability, have contributed to the emergence of chronic inflammatory disorders [47]. The initial stages of carcinogenesis are marked by chronic inflammation, and CRC has long been regarded as one of the greatest instances of malignancies that have a tight relationship with chronic inflammation [48]. NLRP6 is primarily and abundantly expressed in the intestine of mammals, including intestinal epithelial cells and goblet cells, which play a role in the control of intestinal inflammation, the preservation of intestinal mucosal homeostasis, and host defense against microbes [49].

**Figure 2:**
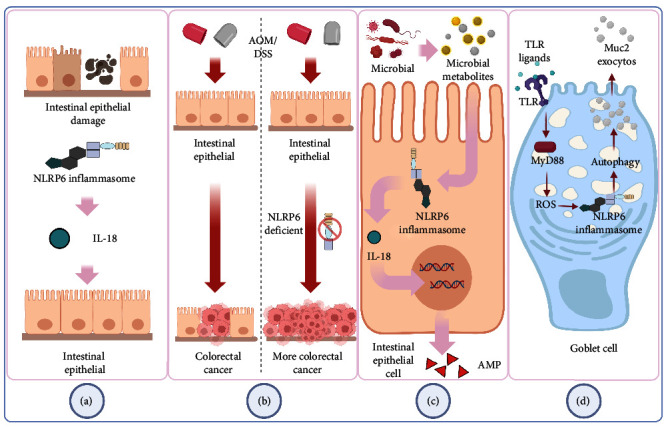
The potential association between NLRP6 and inflammation-related CRC. (a) NLRP6 is involved in maintaining the integrity of the intestinal epithelial barrier. (b) After AOM/DSS treatment, more obvious CAC was induced in NLRP6 gene-deficient mice than in WT mice. (c) The microflora regulates the signal transduction of NLRP6 inflammatory bodies, epithelial IL-18 secretion, and downstream AMP production through related metabolites. (d) In goblet cells, TLR ligands activate the MyD88-ROS pathway to activate NLRP6 inflammatory bodies to promote exocytosis of mucin particles and form a mucous layer of the colon above the epithelial cells. AOM/DSS, azoxymethane/dextran sodium sulfate; CAC, colitis-associated cancer; AMP, antimicrobial peptide; TLR, Toll-like receptor; MyD88, myeloid differentiation primary response-88; ROS, reactive oxygen species.

Inflammation-related CRC is more likely to develop in those with chronic inflammatory bowel disease (IBD) [50]. Leukocyte infiltration, epithelial cell edema, crypt structural abnormalities, and an increase in the space between the base of the crypt and the muscle layer of mucous membrane are the key pathogenic characteristics of human IBD. By giving mice oral dextran sulfate sodium salt (DSS), the researchers created an animal model with histopathological alterations that are comparable to those of human IBD [51]. The function of NLRP6 in maintaining intestinal homeostasis to stop aberrant inflammation and colon tumor formation is highlighted by Chen's work [31]. The NLRP6 gene-deficient mice in Elinav's experiment also exhibited spontaneous intestinal hyperplasia and inflammatory cell recruitment, and after receiving DSS, these mice had more severe colonic inflammation than WT (wild-type) mice [52]. NLRP6 is also crucial in preserving the strength of the intestinal epithelial barrier. The absence of the NLRP6 gene prevents the colonic epithelium's growth and migration after injury, as well as the regeneration of the colonic mucosa. NLRP6 and epithelial self-renewal are related, according to the whole genome expression profile's gene ontology analysis [32] (Figure 2(a)). Intestinal epithelial cell proliferation activity and proliferation time were increased in NLRP6 gene-deficient mice, which may be the cause of promoting tumorigenesis and development. Colitis-associated CRC (CAC) was induced more significantly in NLRP6 gene-deficient mice than in WT mice following treatment with azoxymethane/DSS (AOM/DSS), a common CRC inducer (Figure 2(b)). At various time points, IL-18 levels in the serum and colon tissue of NLRP6 gene-deficient animals decreased, whereas IL-1 was not seen. The underlying cause of this phenomenon may be the drop in IL-18 brought on by the deletion of the NLRP6 inflammasome, whereas the compensatory activation mechanism of the NLRP3 inflammasome in the absence of NLRP6 explains why the level of IL-1 does not fall in this instance [31, 53]. Intestinal epithelial cells may be the primary source of active IL-18 production through NLRP6, according to the results of Elinav's experiment [52]. The fact that IL-18 is a “double-edged sword” that frequently causes inflammation and mediates immune cell infiltration as well as being crucial for intestinal mucosal repair and intestinal barrier function further supports the idea that NLRP6 may exert the protective impact on the intestinal tract through IL-18 [54]. Additional bone marrow chimerism studies demonstrate that NLRP6 in hematopoietic cells prevents carcinogenesis more significantly in the process of NLRP6-mediated resistance to CAC [31]. To promote barrier function and reduce bacterial-driven inflammation and avoid persistent dysfunctional inflammation, inflammatory monocytes recruited after DSS-induced intestinal damage depend on NLRP6 and IL-18 to produce tumor necrosis factor (TNF)-*α* [55]. When intestinal tissue is injured, the hormone IL-22 is released, and while it initially acts as a protective mechanism, if it is not kept under control during the healing process, it will eventually lead to tumor development. Through the NLRP3 or NLRP6 inflammasome, dendritic cells detect damage to the intestinal tissue, which causes them to downregulate the IL-18-dependent IL-22 binding protein (IL-22BP), raising the ratio of IL-22/IL-22BP. The development of colon tumors and the repair of intestinal tissue are both significantly influenced by the IL-22-IL22BP axis [33]. Additionally, in the context of obesity, the downregulation of NLRP6 levels in the colon of CRC patients lowers the integrity of the intestinal barrier, causes local inflammation, and then acts on obese patients' dysfunctional adipocytes, increasing the expression of various inflammasome components and causing an uncontrollable vicious cycle of inflammation that is favorable to the development of tumor microenvironment [35].

Thus, NLRP6 regulates carcinogenesis and inflammation, processes in which hematopoietic and epithelial cells are both involved. Further, more precise studies are required to integrate this inflammatory regulation network and to confirm the precise responsibilities of each component.

Intestinal microbiome components, including particular microorganisms, signaling pathways, and microbiome-associated chemicals, are also intimately linked to CRC [56]. The significance of microflora, or human-related bacteria, in influencing the inflammatory milieu and encouraging tumor growth and dissemination is becoming more and more clear [57]. Through the induction of carcinogenic inflammation and the production of secondary metabolites, intestinal bacteria alter the rate at which CRC progresses [58]. Even between different types of CRC patients, such as between young CRC patients and older CRC patients, there are notable changes in the characteristics of intestinal flora between CRC patients and healthy individuals [59]. NLRP6 is a crucial player in CRC as a potent regulator of gut microbes [60]. It is interesting to note that the absence of NLRP6 in mice colonic epithelial cells also changed the fecal microbiota, with the increase of Bacteroides (Prevodiaceae) and TM7 being the most notable changes [52]. Twenty healthy volunteers and 31 patients with adenoma who had adenoma mucosal biopsy samples had their samples examined. Eight phyla, including Bacteroides and TM7, have significantly different abundances, according to taxonomic analyses [61]. In comparison to WT mice cohabiting with NLRP6 gene defective animals and WT mice raised alone, the NLRP6 gene deletion-induced microbiota plays a significant role in the development of CRC and exacerbates the production of CAC [34]. Based on a comparison of mouse feces, it was discovered that cohabitation altered the intestinal microbiota of WT mice to that of mice lacking the NLRP6 gene [52]. This phenomenon's occurrence is explained as being dependent on the evolution of the microbial community, where NLRP6 plays a role in identification and regulation. Hu's research revealed that the development of CAC is significantly influenced by aberrant microbiota brought on by inflammasomes. The alteration of the intestinal microbiota is facilitated by the deletion of NLRP6 inflammasome. This altered microbiota can not only cause inflammation caused by the chemokine CCL5, locally trigger the IL-6 pathway to promote epithelial growth, but it can also spread to nearby healthy mice [34]. Through related metabolites, including taurine, histamine, and spermine that jointly control NLRP6 inflammasome signaling, epithelial IL-18 secretion, and downstream antimicrobial peptide (AMP) profiles, the microbiome modifies the host–microbiome interface (Figure 2(c)). The destruction of AMP balance and the resulting ecological imbalance is caused by the absence of inflammasome. Following the transplantation of the feces of NLRP6 gene-deficient mice, the intestinal environment of WT mice was taken over by inflammatory-related microorganisms. These microorganisms inhibited the formation of the NLRP6 inflammasome through associated metabolites histamine and spermine, leading to the destruction of metabolite-NLRP6 inflammasome-AMP, which was advantageous to its priority colonization. Normal microbiota can be re-established, and inflammation in the intestine can be reduced by restoring the metabolite-NLRP6 inflammasome-AMP axis [62]. An experiment on *Candida albicans* discovered that *C. albicans*, with or without activity, can suppress human intestinal mucosal barrier function by decreasing the creation of NLRP3 inflammasome and NLRP6 inflammasome, reducing the production of AMP and tight junction proteins, lending further support to this viewpoint [63]. Inflammasome-deficient animals are unable to clear intestinal pathogens from the mucosal surface, leaving them extremely susceptible to prolonged infection, which in turn causes inflammation and ultimately raises the likelihood of carcinogenesis. Additionally, NLRP6 inflammasome is also a crucial coordinator of goblet cell mucin exocytosis, and its absence can result in autophagy abnormalities in goblet cells and reduced mucus output in the colorectal cavity [49] (Figure 2(d)). In acellular microbial viruses like norovirus and rotavirus, NLRP6 binds viral RNA through the RNA helicase Dhx15 and interacts with mitochondrial antiviral-signaling protein (MAVS) to trigger type I/III IFN, which promotes intestinal tissue-specific antiviral response [24] (Figure 1(d)). NLRP6 can significantly reduce intestinal inflammation brought on by viruses. According to several researches, when NLRP6 interacts with dsRNA in vitro and living cells, LLPS takes place. The LLPS of NLRP6 is a typical ligand-stimulated response, and it serves to direct NLRP6 to carry out various tasks depending on the environment of the cell, including antiviral immunity and antibacterial immunity [64]. As a result, using NLRP6's regulatory influence on intestinal microbes effectively can lower the risk of intestinal carcinogenesis. Our current knowledge of NLRP6's function in controlling intestinal flora and reducing the incidence of CRC is deserving of praise, but more animal and human-level related results transformation experiments are still required to be applied as soon as possible in clinical treatment and prevention.

### 3.2. NLRP6 in Hepatocellular Cancer

Hepatocellular carcinoma (HCC), the most prevalent primary malignant liver tumor, is the second most common cancer death cause in the world [65]. The epidemiology of HCC is evolving as a result of growing alcohol use, an increase in the incidence of obesity, and improvements in the treatment of hepatitis B virus and hepatitis C virus infections in recent years. The risk of HCC that is brought on by nonalcoholic steatohepatitis (NASH) and alcohol-related HCC is rising [66]. A documented phase of HCC transition includes the progression from obesity-related nonalcoholic fatty liver disease (NAFLD) to NASH, liver fibrosis, and cirrhosis [67].

NLRP6 connects the microbiota to the pathogenesis of metabolic diseases and systemic self-inflammation, which at first glance appear to be unrelated, and it plays a crucial regulatory function in the linked pathogenic alterations prior to carcinogenesis. By controlling intestinal microbiota, NLRP6 inflammasome, and effector protein IL-18 inhibit the development of NAFLD/NASH and metabolic syndrome. Toll-like receptor (TLR) 4 and TLR9 agonists enter the portal circulation more readily as a result of inflammasome insufficiency, aggravating hepatic steatosis and inflammation. This results in increased expression of TNF*-α* in the liver, which promotes the development of NASH [68]. In mice with fatty liver or steatohepatitis brought on by high-fat diets (HFD) or methionine-choline deficient diets (MCD), the expression of NLRP6 was downregulated. In addition, HFD or MCD diet-induced lipid accumulation and inflammation are also promoted by hepatocyte-specific NLRP6 gene deletion. Finally, it is argued that via blocking the Cd36 and NF-*κ*B pathways, NLRP6 may play a significant role in the pathological development of NASH [69]. NLRP6 also had a lower expression in cirrhotic and fibrotic liver tissues, and in human hepatic stellate cell line LX-2, it had an anti-fibrotic impact by increasing the activity of protein phosphatase magnesium-dependent 1A [70].

Similarly to this, alcoholic hepatitis (AH) is a significant form of alcoholic liver disease (ALD), which is one of the most prevalent chronic liver illnesses in the world and one of the primary causes of liver cirrhosis and liver cancer [71]. Steatosis, inflammation, and fibrosis in the liver were greatly reduced by overexpressing NLRP6 in the AH animal model. By preventing the activation of the NF-*κ*B signal pathway in macrophages during the development of AH, NLRP6 can lower the production of CCL20 and so act as a preventative mechanism [72]. However, in existing studies, NLRP6 does not always show beneficial effects on HCC-related risk diseases. For example, in Lee's NAFLD mouse model, PPAR-*δ* agonists may inhibit the activation of NLRP6 inflammasome and reduce the level of IL-1*β* by regulating AMP-activated protein kinase phosphorylation and reducing reactive oxygen species (ROS) production, and the resulting anti-inflammatory effects may improve liver steatosis in mice [73]. Another study utilizing a mouse model of ALD discovered that inhibiting the activity of the NLRP6 inflammasome significantly decreased the recruitment of liver immune cells and marginally hindered inflammatory signal transduction during ALD, indicating that NLRP6 may contribute to the progression of the condition [74].

In the aspect of HCC, the NEMO^*∆*hepa^ mouse model of spontaneous hepatocellular carcinogenesis was utilized by Schneider to support his hypothesis that NLRP6 deficiency causes gut microbial dysbiosis, which in turn affects the development of HCC. A lack of NLRP6 affects the gut microbiota and the function of the intestinal barrier, making people more vulnerable to intrahepatic microbial translocation. This, in turn, affects the hepatic inflammatory microenvironment, triggers the aggregation of monocytic myeloid-derived suppressor cells and inhibits T-cell function, and promotes the growth of HCC [36]. Liu's work on the topic of HCC looked at the interaction between NLRP6 and fungi in HCC and discovered that an aberrant *C. albicans* colonization rewired the metabolism of HCC and aided in the development of NLRP6-dependent HCC [37]. However, NLRP6 may be a possible tumor suppressor gene that may adversely regulate the E2F and MYC pathways and be linked to a higher degree of immune cell infiltration, resulting in a better prognosis in a pyroptosis risk model that can predict the prognosis of patients with HCC [38]. The reason for the above contradictory conclusions may be due to the different starting points of the researchers, the direction in which the function of the NLRP6 in question is directed, as well as differences in the methods used to build the model and change gene expression as well as the stages of disease evolution. Different inferences can be drawn based on these minute variations, but the connection between NLRP6 and HCC is undeniable. We currently need to define the role of NLRP6 in the occurrence and progression of HCC and comprehend the magnitude of NLRP6's transition between tumor inhibition and cancer promotion.

### 3.3. NLRP6 in Gastric Cancer (GC)

GC is the third most common cause of cancer death worldwide and the fifth most common cancer overall [75]. The stomach and intestine are both digestive organs of the human body, and there are many similarities between them. Both of them have unique ecosystems formed by mucosa and microorganisms, but each has different metabolic regulation combinations [76].

It has been discovered that NLRP6 functions as a tumor suppressor in GC. About 75% of primary GC cases have downregulated NLRP6 expression, and this is directly correlated with the patients' poor prognosis [39]. The signal transducer and activator of transcription 3 (STAT3) signaling pathway's ability to increase cancer cell proliferation, invasion, and the development of chemical resistance in GC is currently well recognized [77]. By controlling STAT3 signal transduction, NLRP6 prevents the migration, invasion, and proliferation of GC cells [41]. It has been established that persistent *Helicobacter pylori* infection is linked to the development of GC [78]. Through AKT/FOXO3 signal transduction, *Helicobacter pylori* can control the expression of NLRP6 and prevent the growth of stomach cancer [41]. In addition, via mediating P14^ARF^-Mdm2-P53-dependent cell senescence, NLRP6 can control the cell cycle of GC cells and further reduce their proliferative potential [39]. In the study Wang, it was found that NLRP6 can interact with glucose-regulated protein (GRP78) through labeled immunoprecipitation and proteomic analysis based on liquid chromatography/mass spectrometry. GRP78 is a heat shock protein. NLRP6 inhibits the growth of GC cells by promoting the ubiquitination of GRP78 [40]. The positive function of NLRP6 in GC is further supported by a study that shows long non-coding RNA (LncRNA) regulates the expression of NLRP6 and takes part in GC. OIP5 antisense RNA1 (lncRNAOIP5-AS1) is a well-known regulator of several different types of malignant cancers. By suppressing NLRP6 expression in GC, it quickens the disease's course [42]. In conclusion, although there are few research on the NLRP6 inflammasome and GC, the significance of NLRP6 in GC is becoming increasingly obvious. It can not only alter the migration and invasion of GC cells but can also control their cell cycle. Clarification is required about the function of NLRP6 inflammasome and NLRP6 in GC.

### 3.4. NLRP6 in Lung Cancer

NLRP6 is frequently discussed in studies about bacterial infection and is characterized in lung studies as a negative regulator of type 2 immune response [79]. NLRP6 has been identified as a key regulator of neutrophil recruitment, generation, and function during bacterial pneumonia and septicemia in the mouse model of pneumonia-derived septicemia caused by Klebsiella pneumonia. It is crucial for the survival of the host, the eradication of bacteria, the inflow of neutrophils, and the creation of the CXC chemokine [80]. NLRP6 plays a role in pulmonary infection by shaping the inflammatory environment.

Nowadays, chronic inflammation has been proven to play a key role in lung tumorigenesis. In the inflammation-related lung cancer model induced by benzo(a)pyrene and lipopolysaccharide, NLRP3 and caspase-1 were colocated in the lung tissue of mice, but this phenomenon was not detected by NLRP6, indicating that the formation of NLRP3-dependent inflammasome promotes the occurrence of pulmonary inflammation-related tumors, but whether NLRP6 is involved in this process remains to be verified [81]. In another experiment on small cell lung cancer (SCLC), NLRP6 was confirmed to be necessary for SCLC-derived exocrine to induce M2 polarization of macrophages and to promote SCLC metastasis [43].

From these two studies, which are the only ones to address the relationship between lung tumors and NLRP6, it can be inferred that NLRP6 may have a role in the development and unfavorable prognosis of lung cancer, which offers a focus for future study. However, further research is required to identify NLRP6's precise role in lung tumors.

### 3.5. NLRP6 in Other Cancers

Other than the colon, liver, and stomach, which have significant levels of NLRP6 expression, NLRP6 is rarely used in cancer research in other organs. The relationship between specific protein 1 and NLRP6 inflammasome in the research of glioma and papillary thyroid cancer (PTC) improves the malignant behavior, immunological avoidance, and radiation resistance of glioma cells [44]. A study that explored the relationship between germline variation of targeted genes and susceptibility to PTC by sequencing found that NLRP6 may be related to susceptibility to PTC [82]. In addition, an analysis containing NLRP6 gene expression was also used to predict the prognosis and anticancer treatment of cutaneous melanoma, confirming the potential link of NLRP6 in melanoma [83].

At present, the reason why there are few studies on the relationship between NLRP6 and tumors in other organs may be due to the low or no expression of NLRP6 and the lack of understanding of NLRP6 in other organs. But at present, the cognition of NLRP6 in these organ diseases is also gradually improving. For example, the research on NLRP6 in the brain is mainly focused on intracerebral hemorrhage (ICH) and cerebral ischemia/reperfusion (I/R). Whether in humans or rats, the expression of NLRP6 is upregulated after ICH. NLRP6 mediates the regulation of autophagy and inflammation in IHC in an inflammasome-dependent manner, resulting in secondary brain injury after ICH [84]. Similarly, in the rat model of middle cerebral artery occlusion/reperfusion, the upregulated expression of Nlrp6 in the brain aggravates the damage of I/R and deteriorates neurological function [85]. In renal studies, the lack of NLRP6 aggravates the severity of the nephrotoxic acute renal injury, which is characterized by increased apoptosis and the pro-inflammatory response of renal tubular cells, indicating that NLRP6 plays a role in renal protection in acute renal injury [86].

It is worth expecting that future studies on the relationship between NLRP6 and tumors will not be limited to these organs. In addition, NLRP3 plays an important role in many kinds of cancers, such as CRC, HCC, GC, head and neck cancer, oral cancer, lung cancer, and so on. Due to the high similarity between NLRP6 and NLRP3, it is believed that the relationship between NLRP6 and tumor will be as clear as NLRP3 in the future [87]. This further shows that we need to speed up the study of NLRP6 and expand the cognitive boundaries of NLRP6 research.

### 3.6. Prospect of Treatment Strategy

The complex mechanism of association between inflammation and cancer offers great possibilities for new cancer prevention and treatment strategies, although clinical studies supporting cancer-related inflammation and innate immune targeting in advanced cancer patients are still in their infancy [14]. According to a vast array of preclinical and epidemiological data, it can be argued that inflammation and innate immunity are significant targets in cancer after the initial success of immunotherapy to control the adaptive immune system [88]. The adaptive immune response is heavily dependent on innate immunity [89]. Therefore, some tumor-promoting effects of inhibiting the innate immune system can be used as a new form of anti-tumor therapy.

Surgery, radiation therapy, chemotherapy, and targeted therapy are examples of traditional cancer treatment approaches [90]. Surgery is most commonly utilized for patients with early-stage tumors when cancer has not yet metastasized, and cancer's boundaries are evident, and it can be used to successfully remove cancer to slow down and treat cancer. Unlike surgery, radiotherapy, chemotherapy, and targeted therapies are designed by interfering with essential cell cycle's events such as DNA, RNA, and its building blocks synthesis, mitotic spindle formation, and specific oncogenic signaling pathways of cancer cells. There is currently evidence that conventional therapies' anticancer effectiveness is also dependent on host innate immunity, specifically innate immunological sensing, and adaptive immunity [91]. Radiation therapy not only relies on local radiation treatment to eradicate or cure malignancies, but it also benefits from innate immunity. Radiation has been found to trigger the immunogenic death of tumor cells and to stimulate the production of DAMPs such ATP, DNA, and RNA, activating host innate immunological sensing pathways like the NLRP3 inflammasome [92, 93]. In contrast, postchemotherapy immune activation in the host has been confirmed by several researchers. Chemotherapy-induced cell death, like radiotherapy, generates DAMPs such as ATP, which activates the NLRP3 inflammasome and boosts tumor-specific T-cell immunity [94]. These intriguing events extend possibilities for clinical treatment methods for NLRP6, and we can incorporate the most relevant regulatory conditions for NLRP6 in standard treatment modalities and construct novel NLRP6 modalities based on traditional treatments.

The predominant form of NLRP6 function is inflammasome. The focus of NLRP6-based treatment techniques will be on therapeutic approaches targeting the NLRP6 inflammasome. First, addressing upstream DAMPs and PAMPs is the most simple and likely most effective treatment technique. Second, we can influence critical inflammasome components such as caspase-1, ASC, pyrin, and others. Finally, two NLRP6 inflammasome-mediated cytokines, IL-1 and IL-18, are deserving of our attention [95]. There are clinical precedents for the use of medications related to each of these features, such as the approval of ibrutinib, an ASC phosphorylation inhibitor, for the treatment of B-cell cancer and Anakinra, an IL-1 inhibitor, for the treatment of rheumatoid arthritis [96, 97]. However, there is still a big undiscovered terrain in cancer that has to be investigated based on the NLRP6 inflammasome, both for the creation of new molecular medicines and for new uses of treatments that are already in clinical use.

NLRP6 is tightly linked to intestinal flora and inflammation, and it plays a role in tumorigenesis by influencing the tumor's inflammatory response, immunological microenvironment, and tumor cell characteristics. Therapeutic methods to regulate intestine microecology are a novel direction for tumor treatment, and studying NLRP6 modulation in combination with modifying intestinal microecological agents may open up new avenues for this therapeutic approach.

Similarly, the association between NLRP6 and several tumors suggests that it may be used as a particular marker for certain tumors, such as colorectal and stomach cancers, to aid in diagnosis.

## 4. Conclusions

NLRP6 is a kind of intracellular PRR, which regulates the production of downstream cytokines through the formation of inflammasome or regulates the immune function in a variety of ways in the form of noninflammasome. People pay more and more attention to the relationship between NLRP6 and different types of tumors, but the specific function of NLRP6 in tumor formation, development, and invasion is still controversial. In the development of CRC, the beneficial role of NLRP6 is commendable. It not only plays an important role in maintaining intestinal homeostasis but also acts as an effective regulator between microorganisms and inflammatory lesions. However, in HCC, the role of NLRP6 is no longer clear. It can not only negatively regulate the impact of intestinal microorganisms on the progression of NAFLD/NASH, reduce the risk of HCC, but also participate in the process of *C. albicans* to accelerate tumor progression. In GC, NLRP6 can inhibit the proliferation of GC cells and inhibit the development of GC. Studies on NLRP6 in other tumors are more scarce, indicating that we need to further study the potential role of NLRP6 in many aspects and levels of tumors.

Furthermore, several indications that NLRP6 contributes to tumorigenesis, whether as a monomer or as an inflammasome, imply that it may be employed as a potentially controlled target for both prevention and treatment.

We need further research to clarify the molecular mechanism and correlation behind NLRP6 transcription, translation, and active inflammasome production, activation, and regulation in cancer and to explore its potential application value in human malignant tumors.

## Figures and Tables

**Table 1:** Role of NLRP6 in cancer development.

Type of cancer	Source of experimental evidence	Outcome	Suggested mechanism	References
CAC	(i) NLRP6^−/−^ mouse	NLRP6-deficient mice have a higher susceptibility to inflammation-induced tumors in the colon	(i) Increased secretion of TNF-*α*, IL-6, and IL-1*β* (ii) Decreased IL-18 production	[31]
	(i) NLRP6^−/−^ mouse	NLRP6-deficient mice have a higher susceptibility to inflammation-induced tumors in the colon	(i) Activation of Wnt signaling pathway	[32]
	(i) IL-22bp^−/−^ mouse (ii) IL-22 ^−/−^ mouse	IL-22/IL-22BP controls tumorigenesis in the colon	(i) NLRP3 or NLRP6 inflammasome perceive intestinal tissue damage leading to IL-18-dependent downregulation of IL-22BP	[33]
	(i) NLRP6^−/−^ mouse (ii) ASC^−/−^ mouse (iii) IL-18^−/−^ mouse (iv) IL-1R^−/−^ mouse (v) IL-6R^F/−^ mice mouse	Altered elements in the microbiota of inflammasome-deficient mice drive inflammatory CAC	(i) Increased IL-6 secretion	[34]

CRC	(i) Wistar rat (ii) Human colorectal adenocarcinoma cell line (HT-29, Caco-2)	Vicious cycle of inflammatory cascade of visceral adipocytes in the context of obesity promotes CRC development	(i) Upregulation of NLRP1, NLRP3, NLRP6, IL-1 *β*, IL-18 in visceral adipose tissue	[35]

HCC	NEMO^*∆*hepa^*/*Nlrp6^−*/*−^ mouse	Gut ecological dysregulation in NLRP6 deficiency impairs antitumor immune surveillance and drives liver disease to cancer	(i) Induction of Toll-like receptor 4-dependent expansion of myeloid-derived suppressor cells in hepatic monocytes (ii) Suppression of T-cell abundance	[36]
	(i) C57BL/6 mouse (ii) NLRP6^−/−^ mouse	Progress of abnormal colonization of HCC by *Candida albicans*	(i) *C. albicans* recodes HCC metabolism via NLRP6	[37]
	(i) HCC tissues and adjacent normal tissues	NLRP6 is associated with higher HCC staging, grading, and poor prognosis		[38]

GC	(i) Human gastric cancer cell line (MKN45, AGS, SGC7901, MGC803) (ii) Human gastric mucosal epithelial cell line (GES-1)	NLRP6 inhibits GC cell proliferation	(i) Activation of P14^ARF^-P53	[39]
	(i) BALB/c mouse (ii) Human gastric cancer cell line (HGC-27, BGC-823, AGS, and 293 T)	NLRP6 has an inhibitory effect on GC cell growth	(i) Promotes the ubiquitination of GRP78	[40]
	(i) BALB/c mouse (ii) Human gastric cancer cell line (MKN-28, BGC-823, AGS, MGC-803, HGC-27, SGC-7901, and MKN-45)	Overexpression of NLRP6 in GC cells leads to a significant decrease in cell proliferation, migration, and invasion, as well as a significant increase in apoptosis	(i) Inhibited the transcription of STAT3 phosphorylation and its target genes Bcl-2 and MMP-2	[41]
	(i) Human GC cell lines (SGC7901, AGS, MKN45, and BGC803)	lncRNA OIP5-AS1 mediates GC progression	(i) Blocking NLRP6 expression	[42]

SCLC	(i) BALB/c mouse (ii) Human SCLC cell line (H446) (iii) Human acute monocytic leukemia cell line (THP-1) (iv) Mouse lung epithelial cell line (TC-1	SCLC-derived exosomes promote SCLC metastasis in vitro and in vivo	(i) SCLC-derived exosomes induce macrophage conversion to M2 type via NLRP6/NF-*κ*B pathway	[43]

Glioma	(i) BALB/c-nu/nu nude mouse (ii) Glioma cell line (U87) (iii) Normal human astrocyte cell line (NAH)	SP1 is associated with proliferation, migration, and invasion of gliomas in vitro and tumorigenesis in vivo	(i) Interaction of SP1 with NLRP6	[44]

CAC, colitis-associated colorectal cancer; IL-22BP, IL-22-binding protein; ASC, apoptosis-associated speck-like protein; CRC, colorectal cancer; HCC, hepatocellular carcinoma; GC, gastric cancer; SCLC, small cell lung cancer; SP1, specific protein 1.

## Abbreviations

NLRP6: NOD-like receptor family pyrin domain containing 6
PRR: Pattern recognition receptors
PAMP: Pathogen-associated molecular patterns
DAMP: Damage-associated molecular patterns
NLR: Nucleotide-binding domain and leucine-rich repeat-containing family
NF-*k*B: Nuclear factor-*κ*B
ASC: Apoptosis-associated speck-like protein
LRR: Leucine-rich repeat domain
NBD: Nucleotide-binding domain
PYD: Pyrin domain
CRC: Colorectal cancer
IBD: Inflammatory bowel disease
CAC: Colitis-associated CRC
AMP: Antimicrobial peptide
HCC: Hepatocellular carcinoma
GC: Gastric cancer.
